# Polyhexamethylene Biguanide:Polyurethane Blend Nanofibrous Membranes for Wound Infection Control

**DOI:** 10.3390/polym11050915

**Published:** 2019-05-22

**Authors:** Anna Worsley, Kristin Vassileva, Janice Tsui, Wenhui Song, Liam Good

**Affiliations:** 1 Royal Veterinary College, Department of Pathobiology and Population Sciences, 4 Royal College Street, London NW1 0TU, UK; Kristin.vassileva.16@ucl.ac.uk (K.V.); lgood@rvc.ac.uk (L.G.); 2 University College London, Centre for Biomaterials in Surgical Reconstruction and Regeneration, Division of Surgery & Interventional Science, 9th floor, Royal Free Hospital, Pond Street, London NW3 2QG, UK; janice.tsui@ucl.ac.uk (J.T.); w.song@ucl.ac.uk (W.S.)

**Keywords:** PHMB, polyhexanide, wound dressing, polyhexamethylene biguanide, polyurethane, electrospinning, nanofibres, antimicrobial, antiseptic

## Abstract

Polyhexamethylene biguanide (PHMB) is a broad-spectrum antiseptic which avoids many efficacy and toxicity problems associated with antimicrobials, in particular, it has a low risk of loss of susceptibility due to acquired antimicrobial resistance. Despite such advantages, PHMB is not widely used in wound care, suggesting more research is required to take full advantage of PHMB’s properties. We hypothesised that a nanofibre morphology would provide a gradual release of PHMB, prolonging the antimicrobial effects within the therapeutic window. PHMB:polyurethane (PU) electrospun nanofibre membranes were prepared with increasing PHMB concentrations, and the effects on antimicrobial activities, mechanical properties and host cell toxicity were compared. Overall, PHMB:PU membranes displayed a burst release of PHMB during the first hour following PBS immersion (50.5–95.9% of total released), followed by a gradual release over 120 h (≤25 wt % PHMB). The membranes were hydrophilic (83.7–53.3°), gradually gaining hydrophobicity as PHMB was released. They displayed superior antimicrobial activity, which extended past the initial release period, retained PU hyperelasticity regardless of PHMB concentration (collective tensile modulus of 5–35% PHMB:PU membranes, 3.56 ± 0.97 MPa; ultimate strain, >200%) and displayed minimal human cell toxicity (<25 wt % PHMB). With further development, PHMB:PU electrospun membranes may provide improved wound dressings.

## 1. Introduction

Preventing and stabilising infection is a global challenge that is growing within healthcare systems. In addition to being a major cause of death, slow healing increases costs and perpetuates patient suffering. For example, one of the most common complications of diabetes mellitus is a chronic diabetic foot ulcer [1]; infection of these ulcers can contribute to serious negative outcomes including limb loss and sepsis [2]. The challenge of infection control is becoming more difficult with rising rates of acquired antimicrobial resistance. This is a worldwide concern that should be taken into consideration in wound infection control. With many populations still overusing antibiotics [3], research into non-antibiotic alternatives is essential. 

Topical antiseptic treatments are commonly used to prevent infection in wounds; they are effective against a wide range of different types of yeasts, fungi and bacteria and result in relatively low levels of antimicrobial resistance [4,5]. There are many different antiseptic treatments currently used in healthcare [5], and many different delivery systems are being investigated. For example, silver nanoparticles have shown promise in hydrogel [6] and microemulsion [7] delivery systems, chlorhexidine in gel form has displayed strong antimicrobial properties [8], and a povidone-iodine foam dressing fully prevented infection in a prospective phase 4 study [9]. Despite comprehensive research on antiseptics for wound infection control, many have undesired side effects or have poor delivery systems: Silver leads to skin sensitisation and has limited tissue penetration; chlorhexidine causes skin sensitisation; iodine-based antiseptics stain the skin and some of the delivery systems for iodine can reduce wound healing or lead to skin sensitisation [4,5,10]. Therefore, there is a requirement for more research on alternative antimicrobials and delivery systems that may provide reduced side effects. 

**Scheme 1:**
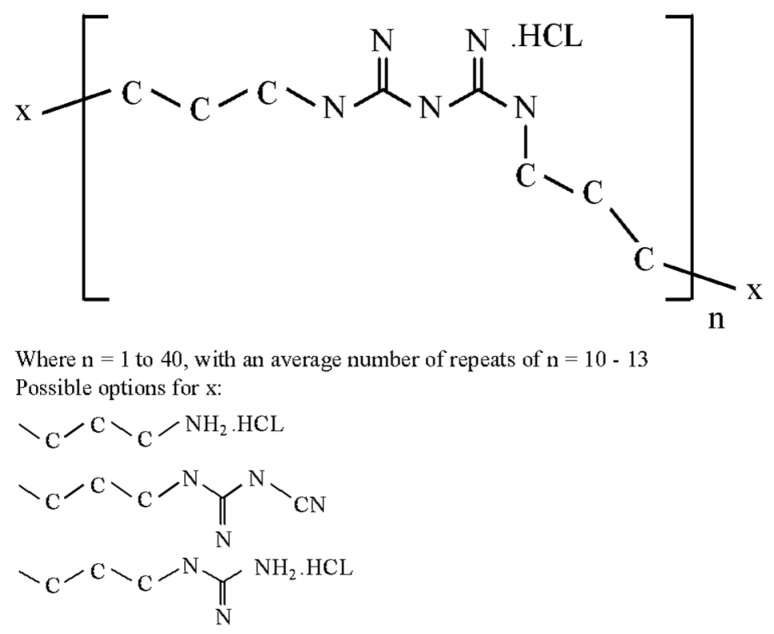
The molecular structure of PHMB. The different variations for end structures (x) and number of repeats (n) are indicated. [12].

Polyhexamethylene biguanide (PHMB), also known as polyhexanide, is a polymerised biguanide compound used as a broad-spectrum antiseptic [4,11], disinfectant [4], and preservative [12]; see Scheme 1. It has proven to be effective against a wide range of pathogens, including strains of *Escherichia coli* [11,13], *Staphylococcus epidermidis* [13,14], and even the Protista *Acanthamoeba castellanii* [14]. It has previously been thought to work primarily through microbial membrane disruption [11,13,15,16], however, more recently it was reported to also selectively bind and condense bacterial DNA, arresting bacterial cell division. This mechanical antimicrobial mechanism of action may help explain why PHMB has a low risk for antimicrobial resistance, of which, none has been recorded despite extensive testing since its first synthesis [11]. Mammalian cells are comparatively unaffected by the polymer as it is segregated into endosomes, seemingly protecting the nuclei from its harmful effects [11], leading to faster wound closures compared to other antimicrobials [17]. 

Many different methods have been evaluated in attempts to improve the release of PHMB [16,18,19]. For example, there is evidence that the method of loading and the use of other substances are important factors for maintaining PHMB’s microbicidal properties [19,20]. Specifically, it has been suggested that antimicrobials in fibrous form have superior release properties [20]. This may be due to an increased surface area, allowing for a high loading capacity and a more gradual release. As a strategy to improve release properties, electrospinning offers a relatively simple and versatile technique to manufacture a range of nanostructured drug delivery systems, from monolithic nanofibres to various multiple drug composition systems [21]. Llorens et al. used electrospinning to fabricate polylactide:PHMB nanofibres [22]. Interestingly, PHMB was only released in the first 1–4 h with a substantial amount not released at all, meaning only higher concentrations of spun PHMB gave sufficient antimicrobial properties when compared to controls [22]. These features indicate a possible route to producing a tunable release of PHMB, where optimisation of the electrospinning process and alternative backbone polymers may improve performance and duration of antimicrobial activity. Despite this, PLA:PHMB electrospun membranes allowed for MDCK cell attachment and were hydrophobic in nature [22]. This may cause cell surface damage when removing the wound dressing [23], as well as not allowing for wound exudate movement and therefore full PHMB release. Hydrophilic polymer matrices, such as polyurethane, may give more beneficial results by avoiding these problems, while matching the mechanical properties of the skin. Polyurethanes have favourable hyperelasticity, strength, wettability and biocompatibility for biomaterial applications [24,25,26,27]. They have been used to incorporate a wide range of compounds, including antimicrobial membranes for wound infection control, and have been intensively characterised [24,25,26,27,28,29,30,31].

This project developed hyperelastic nanofibrous membranes of PHMB and polyurethane (PU) blend through the technique of electrospinning, with the aim of producing an improved antimicrobial wound dressing with a gradual release of PHMB. As the electrospinning of PU with PHMB is unexplored, antimicrobial activity, human cell toxicity and release profile investigations were required. The hypothesis is that electrospinning could be used to engineer the nanostructure of PU-based wound dressings to have high compliance and a sustainable PHMB release overtime to improve antimicrobial effects, with minimal host cell damage. 

## 2. Materials and Methods

### 2.1. Materials

PHMB with an average molecular weight of 3000 g/mol was obtained from Arch Chemicals (Castleford, UK) and Tecrea Ltd., (London, UK). Selectophore™ thermoplastic PU (a medical-grade aliphatic poly(ether-urethane) [25]), 2,2,2-trifluoroethanol, Dulbecco’s Modified Eagle Medium (DMEM), fetal bovine serum (FBS), penicillin/streptomycin, glutaraldehyde, hexamethyldisilazane and ethanol were purchased from Sigma-Aldrich (Dorset, UK). Phosphate buffered saline (PBS) with a pH of 7.4 was purchased from ThermoFisher Scientific (London, UK). Mueller Hinton broth and agar were purchased from Merck (Watford, UK). Alamar blue was purchased from Invitrogen (London, UK). Surgical glue Med1-4013 was obtained from Polymer Systems Technology Ltd. (High Wycombe, UK). Actisorb Silver 220 (Systa genix) was donated by a diabetic foot clinic at the Royal Free hospital, London, UK. HaCaT keratinocytes were obtained from Dr. Amir Sharili, Queen Mary University of London, London, UK.

### 2.2. Preparation of PHMB:PU Nanofibrous Membranes via Electrospinning

#### 2.2.1. Solution Preparation and Electrospinning Optimisation

Eight percent PU (wt %) in 2,2,2-trifluoroethanol was prepared overnight on a magnetic spinner at a temperature of 40 °C. PHMB was then added as a weight percentage (wt %) of PU and stirred at 40 °C for between 30 and 60 min until fully dissolved. A typical vertical electrospinning setup was custom built in house, as described by Wang et al. [25]. For all characterisation experiments, 5, 15, 25 and 35 (wt %) of PHMB were used (5-35PHMB:PU membranes); these were compared to a PU only electrospun control group (0PHMB:PU membranes). High concentrations of PHMB were chosen to optimise the percentage of PHMB released from the wound dressing. Previous research into electrospinning showed that a large quantity of PHMB is trapped within the fibres and is not released [22], suggesting higher concentrations should be included to maintain strong, clinically relevant antimicrobial properties overtime. To find the optimal electrospinning parameters for nanofibrous PHMB:PU membranes, 25PHMB:PU membranes were spun with varying parameters onto glass slides for between 0.5 and 4 min depending on the speed set. These fibres were analysed with a light microscope and tested parameters were chosen in response to previous research papers on electrospinning [32] and initial results. A distance of 17 cm from the collector, a voltage of 25 kV and a flow rate of 2 mL/hour were chosen for all future experiments. 

#### 2.2.2. Sample Preparation

For all experiments, excluding tensile strength tests, electrospinning was carried out for 90 min onto collection platforms (smooth aluminum foil). For tensile strength analyses, samples were spun over 180 min. The edges of all electrospun membranes were then taped down to prevent the material from contracting and creasing while excess solvent evaporated. For PHMB release and biological characterisation studies, the electrospun membranes were cut with a laser cutter (Speedy 100R, trotec, Wells, Austria) into disks with 13-mm diameter. For all other experiments, samples were cut with a scalpel into rectangular shapes. Electrospun samples were not used immediately and contracted and shrunk; disk samples shrunk to an average diameter of 6.84 mm by the time they were tested. These disk samples, used for all release and biological characterisations, weighed an average of 2.30 ± 0.65 mg and contained approximately 0.12, 0.35, 0.58 and 0.81 mg of PHMB in 5-, 15-, 25- and 35PHMB:PU membranes respectively. All samples were stored in normal room conditions. 

### 2.3. Structural, Physical and Mechanical Property Characterisation

#### 2.3.1. Morphology and Fibre Diameter of PHMB:PU Membranes via SEM

A scanning electron microscope (SEM, Carl Ziess EVO HD LS15, Ziess, Oberkochen, Germany) was used with the associated SmartSEM 5.07 software to visualise the morphology of the membranes. Average fibre diameter was then calculated by measuring 30–50 fibre diameters from three different areas of each electrospun membrane. Samples tested included untreated membranes and membranes that had undergone a 24 h immersion in PBS to mimic use in vivo. The latter were washed in distilled water to remove released PHMB that may still reside on the sample surface and air dried before being measured.

#### 2.3.2. Surface Chemistry of PHMB:PU Membranes via ATR-FTIR

Attenuated total reflectance Fourier-transform infrared spectroscopy (ATR-FTIR) was used to assess PHMB levels on the surface of untreated PHMB:PU electrospun membranes and on membranes that had been immersed in PBS for 1 h, 4 h and then every 24 h up to 120 h to mimic use in vivo. These soaked membranes were washed in distilled water and air dried before being measured. Three different material samples were measured for each membrane type. The Jasco ATR-FTIR machine (Jasco, Dunmow, UK) was used for all measurements at room temperature. 

#### 2.3.3. Static Tensile Mechanical Properties of PHMB:PU Membranes

Static tensile strength was measured using an Instron 5565 testing system and Instron Bluehill 3 software (Instron, High Wycombe, UK). The samples were all spun for 180 min to have a similar thickness of 0.17 ± 0.07 mm. Samples were cut with a scalpel to a width of 10 mm and length of 8 mm. Calculations of tensile modulus at 5–10 mm extension, strain at break-up, strength of the membranes and result compilation was done by the software. Tensile toughness was calculated by finding the area under the stress-strain curve. Measurements from three different material samples were taken for each membrane type.

#### 2.3.4. Pore Size and Distribution of PHMB:PU Membranes

The membranes pore size and distribution were characterised by the gas-liquid displacement method using Porolux 1000 (Porometer nv, Nazareth, Belgium). The membranes were cut to have a measurable area of 0.785 cm^2^ and wetted with the specific wetting liquid PorefilL (Porometer nv, Nazareth, Belgium; surface tension of 16 mN·m^−1^). The pressure of the testing gas (N_2_) was increased from 0 to 34.5 bar in a step-by-step manner to replace the wetting liquid inside the pores of each sample. The pressure and flow were stabilised within ±1% for 2 s at each step before the data were recorded. The relevant pore size corresponding to each operating pressure was calculated using the Young-Laplace equation. As well as untreated samples, membranes that had been immersed in PBS for 24 h were tested to mimic use in vivo. These samples were washed in distilled water and air dried before being measured.

#### 2.3.5. Surface Wettability of PHMB:PU Membranes

The contact angle was measured using sessile drop analysis with the DSA 100 instrument and drop shape analysis software (KRUSS, Hamburg, Germany). A flat tip needle with a 0.5 mm diameter was used as part of the syringe pump to dispense the 3 μL volume sessile drop of deionised water. All experiments were undertaken at room temperature. As well as untreated samples, membranes that had been immersed in PBS for 1 h, 4 h and then every 24 h up to 120 h were tested to mimic use in vivo. These samples were washed in distilled water and air dried before being measured. Three different material samples were measured for each membrane type.

#### 2.3.6. PHMB Release Kinetics from PHMB:PU Membranes

**Figure A1:**
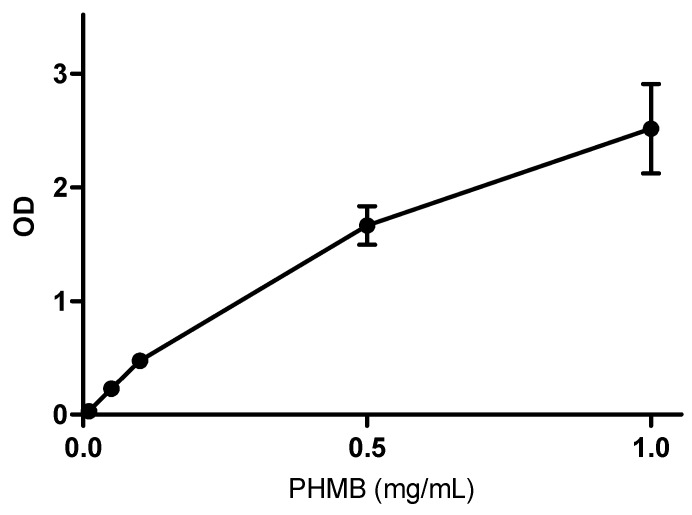
PHMB standard curve in PBS.

The release of PHMB from electrospun membranes was measured using optical density (OD; 230–249 nm [33,34]) readings using the nanoDrop ND1000 spectrophotometer with associated software (version 3.8.1; ThermoFisher Scientific, London, UK). All samples were immersed in PBS in a 96-well plate and left at room temperature. OD readings were taken after 1 h, 4 h and then every 24 h up to 120 h. Before readings were taken, the wells were mixed with a pipette to make sure the released PHMB was evenly distributed for accurate measurements. Concentrations of PHMB released into the liquid were calculated using a standard curve produced using known PHMB concentrations (See Appendix A, Figure A1). Percentage release was then calculated using the known quantity of PHMB within each sample. As well as 5-35PHMB:PU membranes, a non-electrospun PHMB-only control group (ctrl PHMB) was tested. These ctrl PHMB samples were prepared by injecting 20 μL of a 1 mg/mL PHMB/water solution into paper disks with a pipette. These disks were then air dried, forming non-electrospun samples carrying 0.02 mg of PHMB (the equivalent of approximately 1% PHMB when compared to electrospun samples). The ctrl PHMB disks had a similar diameter to the electrospun samples. Three different material samples were measured for each sample type with three measurements taken per sample.

### 2.4. Biological Interaction Characterisations

#### 2.4.1. Antimicrobial Properties of PHMB:PU Membranes

Disk diffusion and liquid bacterial inoculation assays were carried out using *Staphylococcus aureus* RN4220. An overnight liquid bacterial culture was prepared using MH broth, incubated at 37 °C. Samples included the 0-35PHMB:PU electrospun membranes; the non-electrospun PHMB-only controls (ctrl PHMB) as described above in Section 2.3.6; and the commercial wound dressing Actisorb Silver 220 (Systa genix, Skipton, UK), a silver and charcoal woven antimicrobial dressing used in diabetic foot clinics. Three different material samples were used for each sample type. For disk diffusion assays, agar plates were air dried of condensation and evenly inoculated with 1,000,000 colony forming units (CFU) in 100 μL of MH broth from an overnight culture. These were semi-air-dried before the samples were added and then incubated overnight at 37 °C. The zone of growth inhibition that formed overnight was measured and results were normalised by subtracting the disk diameter. For liquid bacterial inoculation assays, samples were incubated in 96-well plates overnight at 37 °C with 280 μL of phenol-free high glucose DMEM containing 1.5 × 10^8^ CFU per mL of *Staphylococcus aureus* RN4220 from an overnight culture. Untreated samples and samples that had been immersed in PBS overnight were tested. After the overnight incubation, 3 × 10 μL diluted samples from each well were seeded onto MH agar plates for analysis. These were incubated for 24 h at 37 °C before the total CFU count for each well was measured. 

#### 2.4.2. Human Cell Viability and Attachment on PHMB:PU Membranes

HaCaT keratinocytes were cultured in low glucose DMEM with phenol red, supplemented with 10% FBS and 1% penicillin/streptomycin in a 37 °C, 5% CO_2_ humidified atmosphere. Samples tested were the same as in the antimicrobial studies undertaken, with tissue culture plate as an extra control group. For SEM analysis, glass controls were also used. Three different material samples were used for each sample type. For toxicity assays, cells were seeded in 12-well plates, with 150,000 cells per well. The first Alamar blue assay was carried out 24 h after seeding. Samples were then added and further Alamar blue assays were carried out after another 24 h and 48 h to assess the toxicity of released PHMB. For each assay, 10% Alamar blue with cell culture media was added to wells and incubated (37 °C, 5% CO_2_ humidified atmosphere) for 4 h before readings were taken. Four × 100 μL samples of the Alamar blue/media were taken per well and placed into 96-well plates. Relative fluorescence readings were taken at 530 nm emission and 620 nm excitation. 

For cell attachment analysis, material was electrospun as described before, but instead of being laser cut, glass coverslips were glued on around its circumference with surgical glue. These were then cut out with a scalpel and sterilised under UV light for 30 min. Cell culture was as described above, but with 1,000,000 cells per well being seeded. After 24 h of incubation, samples were moved to new 12-well plates and incubated in 10% Alamar blue for 4 h before analysis. This experiment was then repeated with samples that had been immersed in cell culture media overnight before cell seeding. For these samples, Alamar blue assays were undertaken 24 h and 48 h after cell seeding. The cells attached to these soaked samples were then fixed for SEM analysis. To fix these samples, membranes were incubated in 2.5% *w*/*v* glutaraldehyde/PBS for 1 h. Samples were then washed in distilled water and dehydrated in a series of ethanol (20%, 40%, 80% and 100% for 10 min each) at room temperature. Samples were then incubated in hexamethyldisilazane at room temperature for 15 min and air dried. Dried samples were sputter coated with 20 nm of gold using a Quorum Q150RS instrument (Laughton, UK).

### 2.5. Statistical Analysis

Statistical significance was assessed using GraphPad Prism 7 software (San Diego, CA, USA). A statistically significant difference was set at *p* < 0.05 (*), *p* < 0.001 (**) and *p* < 0.0001 (***). All error bars are a standard deviation from the mean. One-way Analysis of Variance (ANOVA) tests were used for single time point experiments and two-way ANOVA for multiple time point experiments. 

## 3. Results

### 3.1. Initial Optimisations

As the combination of PU with PHMB had not been evaluated previously, electrospinning parameters were first optimised. Different parameters were compared and the optimal distance between the needle and collector was found to be 17 cm with a voltage of 25 kV and a rate of 2 mL/hour for the most uniform and straight fibres. These parameters were therefore used for all subsequent material production.

### 3.2. Structure, Physical and Mechanical Properties

#### 3.2.1. Morphology and Fibre Diameter of PHMB:PU Membranes via SEM

**Figure 1:**
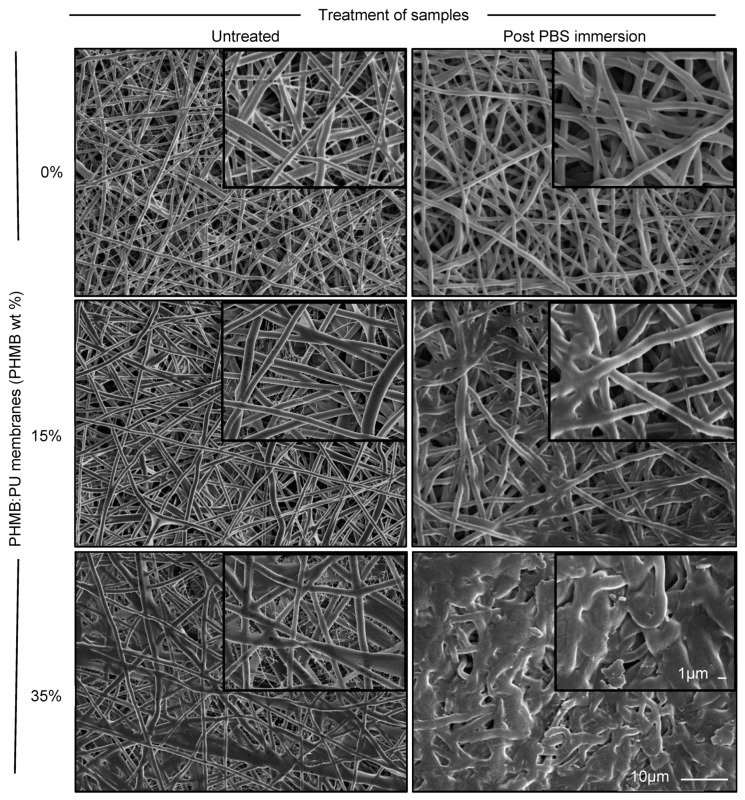
Representative scanning electron microscopy images of 0-, 15- and 35PHMB:PU electrospun membranes. Images are representative of untreated membranes and membranes after immersion in PBS for 24 h. Scale bars are for both untreated and treated samples.

**Figure 2:**
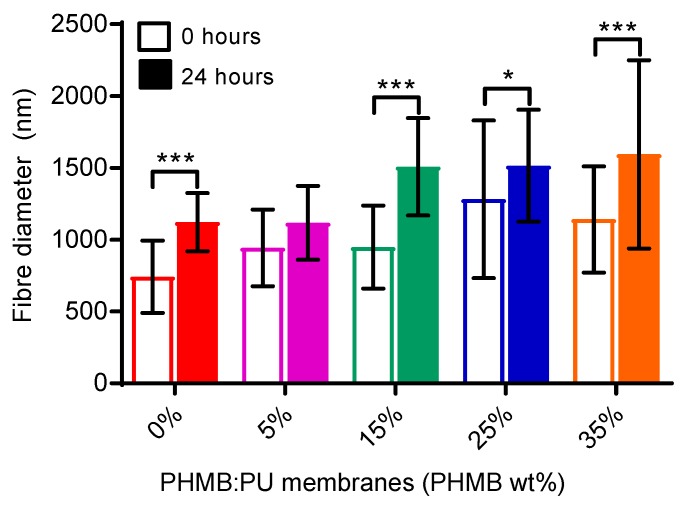
Fibre diameters (nm) of electrospun PHMB:PU membranes. Samples included untreated membranes (0 h) and membranes after immersion in PBS for 24 h. *N* = 20–50, taken from three different areas of material imaged with SEM. Error bars describe the standard deviation from the mean. * signifies a significant percentage change after immersing samples in PBS (* for *p* < 0.05; *** for *p* < 0.0001).

SEM was used to analyse the fibre morphology of untreated electrospun membranes and membranes after a 24 h immersion in PBS to mimic 24 h of use. Fibres of electrospun PHMB:PU membranes before treatment were long, straight and uniform. However, with increasing amounts of PHMB they became thicker and more sinuous (Figure 1 and Figure 2, 0 h). There was a gradual increase in fibre diameter as PHMB concentration increased, however there were no immediate significant differences between PHMB:PU membranes, see Figure 1 and Figure 2, 0 h. All membranes also had thinner web-like fibres in-between the main fibres, these were found to be much thinner in diameter (52 ± 21 nm in all membrane samples). 

After a 24-h immersion in PBS, fibres in all membrane samples became less uniform and distinct. This change was more prominent with higher PHMB contents (Figure 1, 24 h). Fibres with a lower concentration of PHMB (<25%) become more sinuous after immersion, but retained their fibrous structure. In 35PHMB:PU membranes, the fibres were severely deformed and adhered together, with the apparent closure of pores. Immersion in PBS also caused fibres to swell, with fibre diameters increasing by 51%, 18.4%, 59%, 18.1% and 40% in 0-35PHMB:PU membranes respectively after 24 h (Figure 2, *p* < 0.0001 for 0-, 15- and 35PHMB:PU membranes, *p* < 0.05 for 25PHMB:PU membranes). The relatively large standard deviation for all sample measurements reflects a relatively wide distribution of the fibre diameters in both untreated and treated membranes (Figure 2).

#### 3.2.2. Surface Chemistry of PHMB:PU Membranes via ATR-FTIR

**Figure 3:**
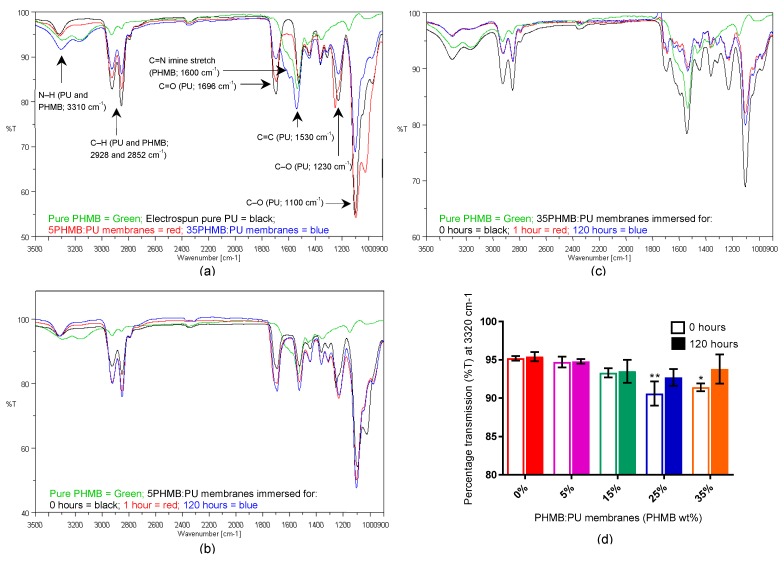
FTIR measurements of PHMB:PU electrospun membranes. (**a**) Pure PHMB (green), untreated membranes: 0-, 5- and 35PHMB:PU electrospun membranes (black, red and blue respectively). (**b**,**c**), 5- and 35PHMB:PU membranes respectively that were immersed in PBS for 0, 1 and 120 h (black, red and blue respectively). These were compared to pure PHMB (green). (**d**) Percentage transmission for the 3320 cm^−1^ peak (N–H) before and after membranes were immersed in PBS for 0 and 120 h. * indicates a significant difference (* for *p* < 0.05; ** for *p* < 0.001).

The FTIR spectra for electrospun PHMB:PU membranes had characteristic absorption peaks for PU (functional groups of N–H, C–H, aromatic C=C and ester groups C=O and C–O at wavelengths of 3320, 2928 and 2852, 1530, 1696, 1230 and 1100 cm^−1^ respectively [25]) and PHMB (functional groups of C=N, N–H and C–H with wavelengths of 1600 cm^−1^ [stretch], 3310 cm^−1^, 2928 and 2852 cm^−1^ respectively [35]), see Figure 3a–c. Percentage transmission at these wavelengths exhibited a trend of PHMB influence on PU peaks as PHMB levels increased (Figure 3). In particular, the stretch corresponding to PHMB’s imine group became more pronounced as PHMB concentration increased (Figure 3a–c). The amine peak (N–H) characteristic of PU and PHMB also had a dampened percentage transmission with the addition of PHMB (Figure 3a–c), significantly decreasing for 25- and 35PHMB:PU membranes before PBS immersion (Figure 3d, 0 h). This is likely related to a secondary peak forming at around 3150 cm^−1^ with higher PHMB concentrations, corresponding to PHMB’s primary amine groups as opposed to PU which only has secondary amine groups. 

#### 3.2.3. Tensile Mechanical Properties of PHMB:PU Membranes

**Figure 4:**
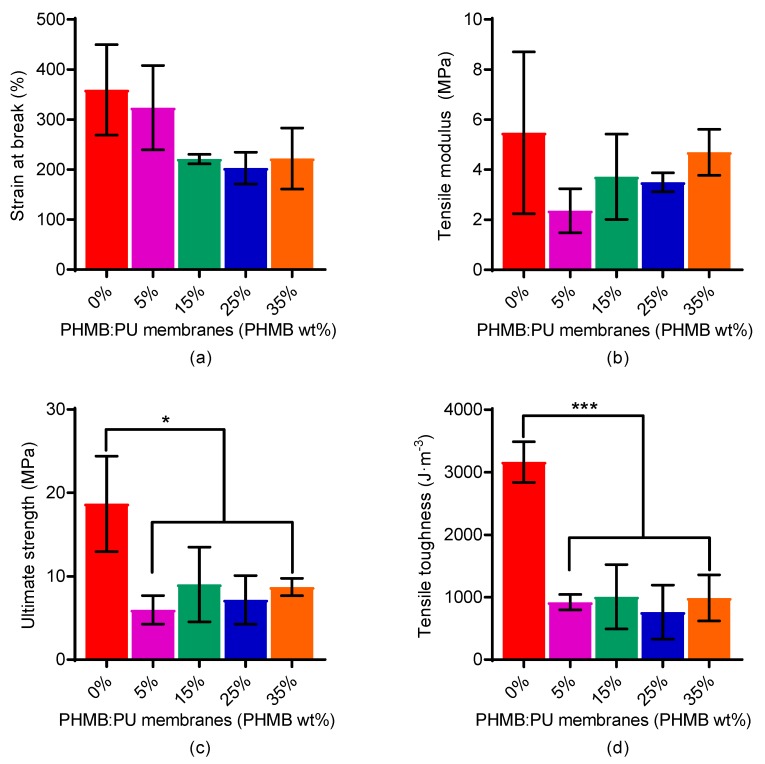
Tensile mechanical properties of electrospun PHMB:PU membranes. (**a**) Strain at break; (**b**) Tensile modulus at 5–10 mm extension; (**c**) Ultimate strength; (**d**) Tensile toughness. Samples tested were the 0-35PHMB:PU electrospun membranes. Error bars describe the standard deviation from the mean, with *n* = 3. * signifies a significant difference between samples (* for *p* < 0.05; *** for *p* < 0.0001).

The overall tensile mechanical properties of PHMB:PU membranes, in terms of the strain at break, elastic modulus, ultimate strength and toughness, were reduced in the presence of PHMB, compared to pure PU electrospun membranes (0PHMB:PU), as shown in Figure 4. In particular, the ultimate strength and tensile toughness of PHMB:PU electrospun membranes were seen to decrease significantly by an average of 58.65% and 70.95% respectively when compared to electrospun PU only membranes (Figure 4c,d, *p* < 0.05 and *p* < 0.001 respectively). However, this trend did not reflect the increasing load of PHMB to electrospun PU membranes as no significance was recorded between 5-35PHMB:PU membranes.

#### 3.2.4. Pore Size and Distribution of PHMB:PU Membranes

**Figure 5:**
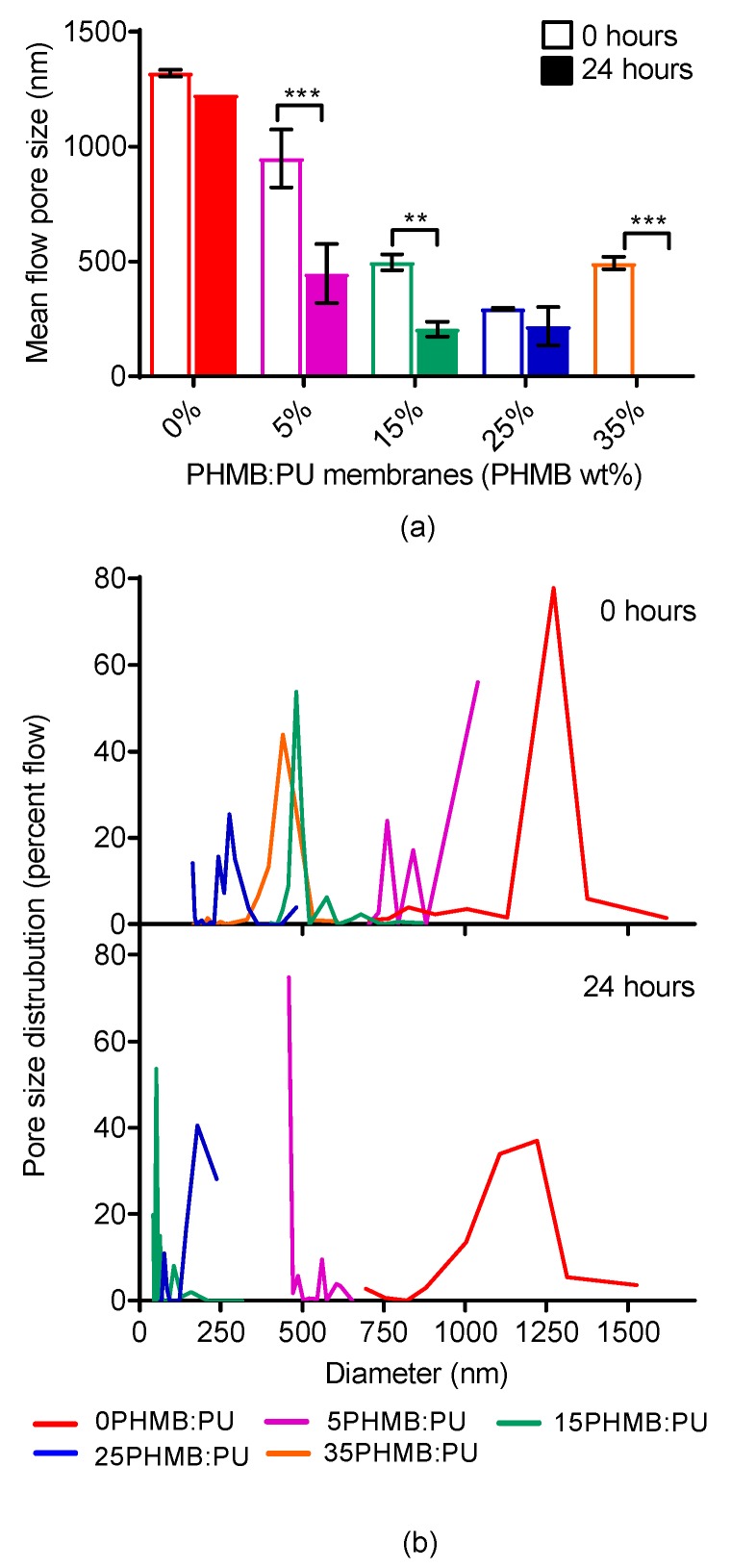
The effect of increasing PHMB concentration on pore size in PHMB:PU electrospun membranes. (**a**) Mean flow pore size (nm). Error bars describe the standard deviation from the mean, with *n* = 20–114. * signifies a significant percentage change after immersing samples in PBS (** for *p* < 0.001; *** for *p* < 0.0001). (**b**) Representative pore size distributions (percentage flow of N2) after a 0-h and 24-h immersion in PBS. 35PHMB:PU membranes were not included in (**b**), 24 h, as they were recorded as impermeable.

Pore size and distribution were evaluated to access the breathability and permeability of membranes. As the content of PHMB increased, the pore size of the membranes reduced drastically, but were not fully exhausted (significant differences recorded between 0-, 5- and 15-35PHMB:PU membranes, Figure 5). After the PHMB:PU membranes were immersed for 24 h in PBS, pore size decreased further with 35PHMB:PU membranes becoming impermeable. 

#### 3.2.5. PHMB Release Kinetics of PHMB:PU Membranes

**Figure 6:**
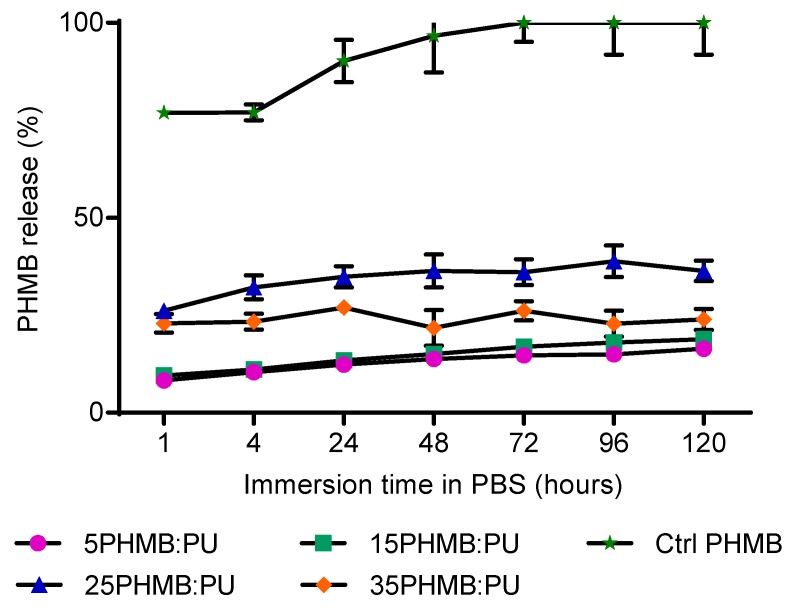
Average percentage release of PHMB from electrospun PHMB:PU membranes after immersion in PBS. Error bars describe the standard deviation from the mean, with *n* = 3. Paper disks freely infused with PHMB (Ctrl PHMB) were used as controls, as described in the methods section.

To prove the hypothesis that an electrospun nanofibrous structure would allow for a gradual delivery of PHMB, the PHMB:PU membranes were immersed in PBS and the PHMB release was measured over time. All membranes showed a significant burst release of PHMB within the first hour; see Figure 6 (*p* < 0.05 for 5PHMB:PU membranes, *p* < 0.001 for 15PHMB:PU and *p* < 0.0001 for 25-35PHMB:PU membranes). The percentage of the total released PHMB released in this initial burst increased as PHMB concentration increased from a 15% content (after 1 h, 5-35PHMB:PU membranes released 50.5%, 50.4%, 71.9% and 95.9% of their total PHMB content released respectively). The initial burst release was followed by a more gradual release of PHMB in 5-25PHMB:PU electrospun membranes (no significant differences between each immediately succeeding time point, but significant differences measured between 1 h and 120 h time points, *p* < 0.0001). In contrast, 35PHMB:PU electrospun membranes showed no further significant release after the first hour. All electrospun membranes released a significantly lower percentage of their total PHMB content compared to non-electrospun PHMB samples, reflecting work done by Llorens et al. who electrospun PHMB with polylactide [22], (*p* < 0.0001, Figure 6; percentage released at 120 h: 16.4% ± 0.69% from 5PHMB:PU membranes, equating to an average of 0.02 mg PHMB; 18.8% ± 1.2% from 15PHMB:PU membranes, equating to an average of 0.07 mg PHMB; 36.3% ± 2.7% from 25PHMB:PU membranes, equating to an average of 0.2 mg PHMB; 23.9% ± 2.7% from 35PHMB:PU membranes, equating to an average of 0.2 mg PHMB).

#### 3.2.6. Surface Wettability of PHMB:PU Membranes

**Figure 7:**
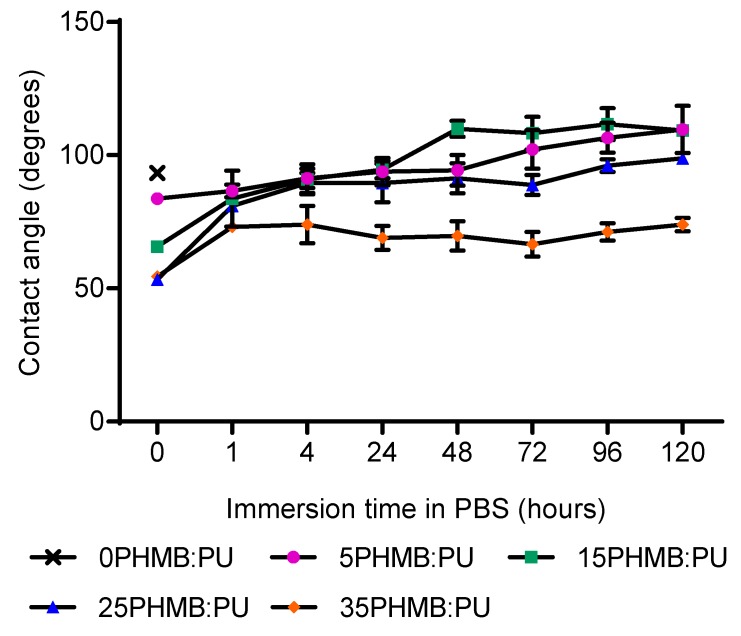
The contact angle change of electrospun PHMB:PU membranes after immersion in PBS. Changes in contact angle inversely indicate changes in wettability as PHMB was released. Error bars describe the standard deviation from the mean, with *n* = 3. 0 h measurements for 35PHMB:PU membranes were taken but were found to give unstable droplet formation, indicating higher hydrophilicity than reported.

As PHMB content increased in the PHMB:PU membranes, surface hydrophilicity increased (as seen in Figure 7, 0 h measurements; the contact angles for 0-35PHMB:PU electrospun membranes were measured at 93° ± 2°, 84° ± 1°, 66° ± 1°, 53° ± 1° and 55°± 1° respectively, significant differences between all values excluding between 0- and 5PHMB:PU membranes and between 25- and 35PHMB:PU membranes, *p* < 0.05). 35PHMB:PU membranes at 0 h were measured to have a contact angle similar to that seen for 25PHMB:PU membrane, however, the droplets were unstable and dissipated after a few minutes, indicating higher levers of hydrophilicity than measured. 

The contact angle of electrospun PHMB:PU membranes was recorded after membranes had been immersed in PBS, revealing the change in wettability as PHMB was released. 35PHMB:PU membranes had a significant decrease in wettability in the first hour, which then stabilised and remained constant over the next 119 h analysed. 15- and 25PHMB:PU electrospun membranes also showed this initial significant decrease in wettability (the surface contact angle of 15-, 25- and 35PHMB:PU membranes increased by 27.39%, 51.89% and 34.21% respectively after 1 h of PBS immersion, *p* < 0.0001, Figure 7). However, after this first hour, 5-25PHMB:PU electrospun membranes had a more gradual decrease in wettability up to the 120 h time point (significant increases in contact angle recorded between 1 and 120 h but not between each immediately succeeding time point; the overall increase of surface contact angle for 5-, 15- and 25- PHMB:PU membranes was 26.73%, 30.54% and 21.98% respectively, *p* < 0.001). After 120 h, wettability of 5-25PHMB:PU electrospun membranes decreased to values similar to PU only membranes (0PHMB:PU, Figure 7). 

### 3.3. Effects on Pathogen and Host Cells

#### 3.3.1. Antibacterial Activities of PHMB:PU Membranes

**Figure 8:**
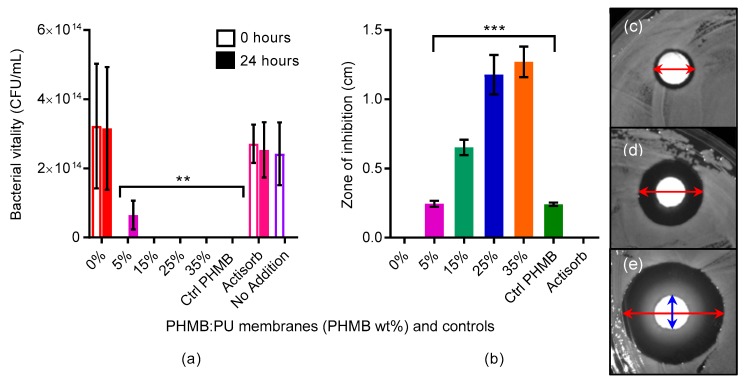
Antibacterial activities of PHMB:PU membranes. (**a**) Antimicrobial activity in liquid *S.aureus* RN4220 cultures. Surviving bacterial colony forming units (CFU/mL) after overnight incubation with samples. Samples included PHMB:PU electrospun membranes and controls before (0 h) and after a 24 h immersion in PBS. (**b**) Zones of bacterial (*S.aureus* RN4220) growth inhibition using disk diffusion assays. Error bars describe the standard deviation from the mean, with *n* = 3. * indicates a significant difference to 0% electrospun membrane samples (** for *p* < 0.001; *** for *p* < 0.0001). Controls used were PU only (0PHMB:PU membranes), PHMB freely infused in paper (Ctrl PHMB), the commercial product Actisorb Silver 220 (Actisorb), and for (**a**), no addition of material or reagent to bacterial liquid cultures. Images (**c**–**e**) are representative of the zones of bacterial growth inhibition measured as shown in (**b**), (5-, 15- and 35PHMB:PU electrospun membranes respectively). The red arrows indicate the inhibition zone diameter measured, this was normalised by subtracting the sample diameter (**e**, blue arrow).

The antimicrobial activity of the PHMB:PU membranes was first examined using disk diffusion assays, where sample disks were placed on lawns of bacteria and the diameter of growth inhibition was measured after 24 h. Antimicrobial activity increased as PHMB content increased, with all PHMB:PU membranes having a significantly higher antimicrobial activity than PU only membranes, as shown in Figure 8b. 5PHMB:PU membranes showed similar antimicrobial activity compared to control PHMB paper disks despite containing more PHMB, indicating a gradual release of PHMB from 5PHMB:PU membranes (Figure 8b). 

Measurements from liquid inoculation incubations, where samples were suspended in infected liquid broth, also displayed impressive antimicrobial activity (Figure 8a); 0 CFU/mL survived after 24 h of incubation with all PHMB:PU electrospun membranes. After 24 h of immersion in PBS, electrospun 15-35PHMB:PU membranes showed a continuation of their antimicrobial activity strength. 5PHMB:PU electrospun membranes lost some of their antimicrobial activity after the 24 h of immersion, although this was not statistically significant; the level of antimicrobial activity was still significantly lower than 0PHMB:PU groups (*p* < 0.05) and was not significantly different to 15-35PHMB:PU membranes (Figure 8a). Control PHMB samples still showed antimicrobial activity after 24 h of immersion, however this was seen as a false positive result. Unlike the PHMB:PU membranes, the paper control disks could not be efficiently washed to remove excess PHMB collected on the surface because it had partially deteriorated with immersion, allowing for released PHMB to collect and remain on the sample surface after removal from the PBS. 

#### 3.3.2. HaCaT Cell Responses

##### Human Cell Toxicity of PHMB:PU Membranes

**Figure 9:**
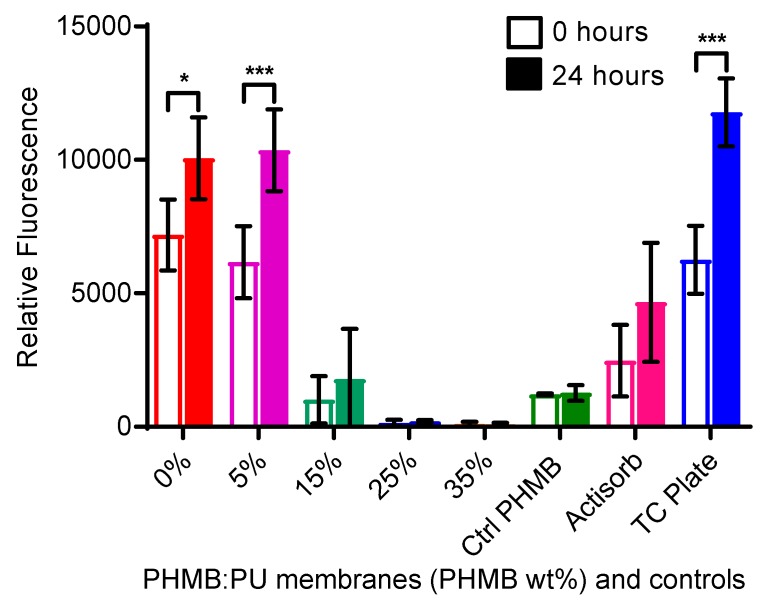
HaCaT cell toxicity. Alamar blue results taken 24 and 48 h after sample addition. Controls included TC plate (tissue culture plate), paper disks freely infused with PHMB (Ctrl PHMB) and the commercial product Actisorb Silver 220 (Actisorb). Error bars are the standard deviation from the mean, with *n* = 3. * indicates a significant difference between time points within samples groups (* for *p* < 0.05 and *** for *p* < 0.0001 respectively).

Alamar blue assays were used to assess the toxicity of PHMB:PU electrospun membranes against cultured human keratinocytes via metabolic activity. 5PHMB:PU electrospun membranes showed no cell toxicity at all time points (Figure 9). The same was seen for pure PU only electrospun membranes, displaying the non-toxic nature of PU. After 24 h of incubation with samples, the cell viability levels of 15PHMB:PU membranes were not significantly different to the commercial product Actisorb Silver 220 or the non-electrospun PHMB control groups, all of which showed a significant amount of cell toxicity (Figure 9, *p* < 0.05). However, after 48 h, Actisorb Silver 220 showed slight but significant improvements in cell viability, while 15PHMB:PU membranes did not. Finally, 25- and 35PHMB:PU electrospun membranes showed high levels of toxicity that were not recoverable during the monitoring period. 

##### Human Cell Attachment on PHMB:PU Membranes

**Figure 10:**
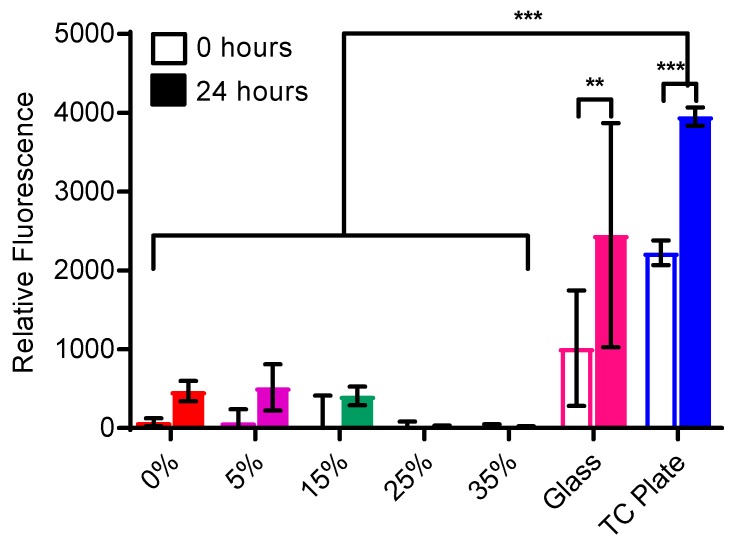
HaCaT cell metabolic activity on PHMB:PU membranes. Cell metabolic activity after 24 and 48 h using the Alamar blue assay on membranes which had been immersed in cell culture media overnight before cell seeding to mimic attachment after use in vivo. Tissue culture plate (TC plate) and glass were used as controls. Error bars describe the standard deviation from the mean, with *n* = 3. * indicates a significant difference (** for *p* < 0.001; *** for *p* < 0.0001).

**Figure 11:**
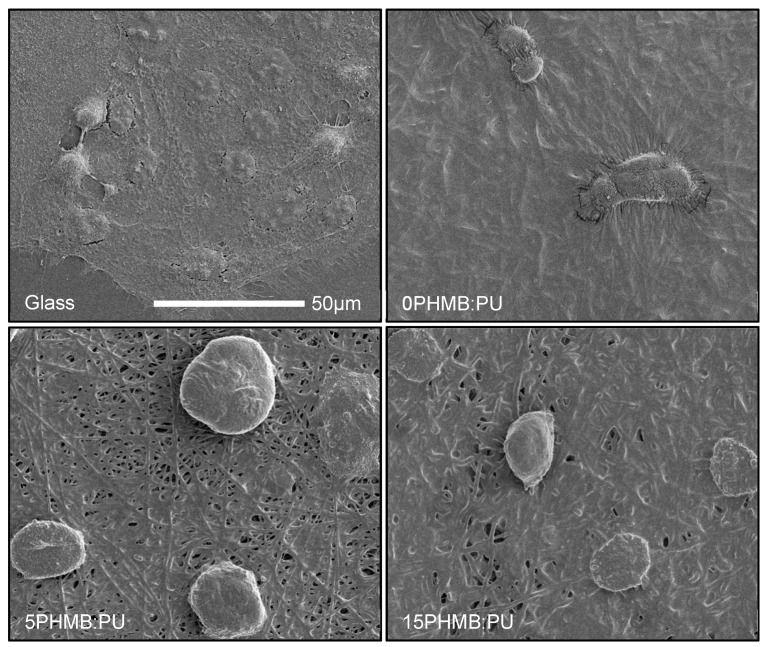
HaCaT cell morphology on PHMB:PU membranes. SEM images showing cell morphology after 48 h on membranes which had been immersed in cell culture media overnight before cell seeding to mimic attachment after use in vivo.

All electrospun PHMB:PU membranes showed no successful cell attachment after 24 h of incubation. Cells were then seeded onto PHMB:PU electrospun membranes that had been immersed in cell culture media for 24 h to allow for more protein attachment and to mimic use overtime. Some attachment was seen with these samples (Figure 10). However, as seen in the SEM images, these cells were rounded in morphology showing unfavorable attachment conditions (Figure 11). 25- and 35PHMB:PU electrospun membranes showed minimal cell attachment but showed high levels of toxicity in the previous section; therefore, they could not be imaged.

## 4. Discussion

PHMB was successfully incorporated within PU nanofibre membranes at concentrations ranging from 5% to 35% (Figure 3). The aim of this project was to develop a wound dressing which would allow PHMB to be released in a gradual manner, allowing for the optimisation of PHMB’s antimicrobial properties. This was accomplished using 5-25PHMB:PU membranes; when immersed in PBS, these membranes had an initial burst release of PHMB followed by a more gradual release over the 120 h measured (Figure 6). This was reflected in the change of wettability also recorded with PBS immersion overtime (Figure 7). The initial burst release measured within the first hour mirrors similar results recorded by Llorens et al. [22] and could be beneficial for heavily infected wounds. The gradual release after the initial burst would prevent bacteria growth recovery and colonisation overtime, providing ideal conditions to reduce bacterial load while minimising host cell toxicity. The frequency of wound dressing changes may also be reduced as the PHMB:PU membranes retain their antimicrobial activity overtime [36]. 

5-25PHMB:PU membranes were found to be porous even after use (Figure 5), allowing for breathability and permeability for wound exudate passage. Fibres became swollen and more sinuous when immersed in PBS (Figure 1), causing pores to shrink but not fully close up. This change in fibre diameter and morphology may be due to the hydrophilic nature of PHMB, which could promote some liquid absorption prior to PHMB release and is consistent with the observed increase in wettability (Figure 7). Along with the hydrophilic nature of the PHMB:PU membranes (Figure 7), the continued permeability would aid continued PHMB release and may allow for a moist wound bed that does not become overly wet, aiding wound healing further [36]. The decrease in pore size with increasing PHMB content also displays a possible route for tunability. 

The addition of PHMB to PU electrospun membranes slightly reduced their elasticity and tensile strength and significantly decreased their ultimate strength and tensile toughness (Figure 4). This suggests that PHMB:PU electrospun membranes are elastic but are quicker to tear with deformity than with pure PU electrospun membranes. Despite this, the overall tensile strength, modulus and ultimate strain of the blend membranes remained favorable for a wound dressing as they still retained the hyperelastic and durable properties of polyurethane that are relevant to skin [37].

As 5PHMB:PU electrospun membranes showed no cell toxicity and 15PHMB:PU membranes had comparable toxicity levels to non-electrospun PHMB samples, despite a higher PHMB content (Figure 9), these samples are the most promising for further development. Cell death in response to 25- and 35PHMB:PU membranes was possibly because too much PHMB released in the first burst; therefore, the amount of PHMB entering the cells was too high for them to cope leaving some to enter the nuclei and condense chromosomes, as seen in bacterial cells [11]. These higher concentration membranes are unlikely to be used in future work unless the release is slowed in further material optimisations. 

Cell attachment and cell morphology analysis indicated that PHMB:PU membrane surfaces were not ideal for cell attachment (Figure 10 and Figure 11 respectively). This may be related to the high positive charge of PHMB causing some abnormal protein attachment; when released onto the membrane surface, proteins may bind too strongly, resulting in abnormal conformation and protein denaturation. It is important to clarify that low cell attachment is in fact beneficial for wound dressings as it would prevent healthy tissue removal on dressing changes [23]. Therefore, our results suggest that PHMB:PU electrospun membranes could avoid associated complications with healthy cell removal, improving the wound healing process. 

## 5. Conclusions

Electrospun PHMB:PU nanofibrous membranes offer an exciting alternative strategy for providing a gradual release of PHMB in wound infection prevention and control, and show promise for the development of a future antimicrobial wound dressing. Their elastic and hydrophilic nature could aid patient comfort and help prevent host cell damage. The strong antimicrobial properties sustained overtime may provide an ideal dressing for clearing bacterial load while enhancing healing. Sustained PHMB release may also mean fewer wound dressing changes, saving time, waste products and money. For future work, the tunability of PHMB:PU membranes will be further investigated to produce an optimised wound dressing material.
